# Poster Session I – A97 BARRIERS AND FACILITATORS TO GLUTEN-FREE DIET ADHERENCE IN ADULTS WITH CELIAC DISEASE: A QUALITATIVE STUDY

**DOI:** 10.1093/jcag/gwaf042.097

**Published:** 2026-02-13

**Authors:** V Noejovich, S Jack, P Miranda, M Maarza, D Armstrong, M Pinto-Sanchez

**Affiliations:** Medicine, McMaster University, Hamilton, ON, Canada; Medicine, McMaster University, Hamilton, ON, Canada; McMaster University, Hamilton, ON, Canada; Medicine, McMaster University, Hamilton, ON, Canada; Medicine, McMaster University, Hamilton, ON, Canada; Medicine, McMaster University, Hamilton, ON, Canada

## Abstract

**Background:**

A strict gluten-free diet (GFD) is the only effective treatment for celiac disease (CeD); however, maintaining adherence can be challenging for many adults. Barriers vary across populations and settings. Understanding these factors is essential to improve adherence and optimize clinical outcomes in CeD.

**Aims:**

To identify factors influencing adherence to a GFD among adults with CeD and to explore the care and support needs that healthcare teams should address to improve adherence.

**Methods:**

A qualitative study was conducted using semi-structured interviews with adults (18–75 years) with biopsy-confirmed CeD who had followed a GFD for at least 30 days. Purposive sampling ensured variation in GFD duration and self-reported adherence. Interviews were conducted virtually, audio-recorded, and transcribed verbatim. Data were managed in NVivo and analyzed using reflexive thematic analysis. Two researchers independently coded transcripts to identify key themes and subthemes, resolving discrepancies through discussion to ensure analytical rigour.

**Results:**

From July 2024 to June 2025, 38 participants were enrolled (83% female; mean age 37 years). Most (97.6%) reported strict GFD adherence, and 87.8% had followed the diet for over one year. Participants described multiple, interrelated factors affecting adherence. Social pressures, cross-contamination risks, cravings, and financial or workplace constraints were frequent challenges. Supportive family and friends, effective planning, flexible work arrangements, and adequate income facilitated adherence. Cultural barriers emerged when traditional foods were difficult to adapt, while supportive hosts and adaptable recipes promoted inclusion. 73.7% of participants have experienced some form of stigma related to GFD. Limited access to trained dietitians and inconsistent guidance were key healthcare barriers, whereas multidisciplinary care, practical education, and peer support were strong facilitators.

**Conclusions:**

Adherence to a GFD in CeD is shaped by social, personal, financial, cultural, and healthcare factors. Strengthening family, workplace, and healthcare support—particularly through improved dietitian access and practical education—may enhance adherence and long-term disease management.

**Funding Agencies:**

Balsam Douglas Foundation

**Table:** 

Domain	Challenges (examples)	Facilitators (examples)
Social & family	Eating at others’ homes; social pressure; cross-contamination risk when eating out	Supportive family & friends; dedicated prep areas.
Personal factors	Picky eater; people pleaser; cravings, self-control fatigue	Planning habits; self-control & discipline; symptom-linked motivation
Work settings	Limited safe options at work/events; Cross contamination in shared kitchens	Flexible work arrangements; bringing backup food; supportive coworkers and employers
Financial	Higher cost of GF products; budget constraints & trade-offs	Having good income & secure job; tax rebates.
Cultural	Traditional dishes difficult to adapt; not able to share at family gatherings	Easy to adapt traditional recipes to GF; supportive hosts
Health-system	Limited access to trained dietitians; inconsistent guidance	Dietitian support; multidisciplinary follow-up; practical education; peer support network

